# Can multidisciplinary teams improve the quality of primary care? A scoping review

**DOI:** 10.1016/j.eclinm.2025.103497

**Published:** 2025-09-12

**Authors:** Shona Marie Bates, Jialing Lin, Luke N. Allen, Michael Wright, Michael Kidd

**Affiliations:** a International Centre for Future Health Systems, University of New South Wales, Sydney, Australia; b School of Population Health, University of New South Wales, Sydney, Australia; c Nuffield Department of Primary Care Health Sciences, University of Oxford, Oxford, United Kingdom; d Global Primary Care and Future Health Systems, Nuffield Department of Primary Care Health Sciences, University of Oxford, Oxford, United Kingdom

**Keywords:** Primary care, Multi-disciplinary teams, Multi-disciplinary care, Health system reform

## Abstract

**Background:**

There is increasing use of multi-disciplinary teams (MDT) to increase the quality of and access to primary care. For example, the promotion of MDTs in primary care is one of four recent major policy recommendations in Australia. This review sought to understand the impact of MDT on the quality of primary care, including continuity of care, and the enablers and barriers to implementation.

**Methods:**

A scoping review was undertaken of peer-reviewed journal articles published between 1 January 2014 and 13 August 2024. It is registered at OSF: DOI:10.17605/OSF.IO/23QYU. A search of PubMed, Cochrane, Embase, CINAHL, PAIS, Web of Science, PsycINFO, and Scopus databases yielded 1603 records or 770 articles after duplicates were removed; 75 full-texts were reviewed and 27 studies met the inclusion criteria. The search was repeated for the period 13 August 2024 to 13 August 2025, which yielded a further 282 records after duplicates were removed; 19 full-texts were reviewed and an additional 12 papers met the inclusion criteria reflecting the increasing interest in MDT-care in primary care reforms. Data extracted from the 39 papers in scope included the characteristics of MDT care reported, the outcomes observed, and the enablers and barriers to implementation. A socio-ecological model was used to examine the system, organisational, professional and patient level factors that enabled MDT-care in general practice.

**Findings:**

Data showed the models of MDT-care varied substantially. They ranged from multiple providers working together to care for a patient, to interprofessional teams providing patients the option to see an alternative provider. Analysis showed mixed outcomes from MDTs in primary care, driven by contextual, policy, organisational, professional and patient factors. In some cases, MDT strengthened the management of chronic disease. In other cases, MDT reduced continuity of care by fragmenting relational continuity. MDT care also impacted access to care, comprehensiveness of care, and coordination of care—in some cases positively, and other cases negatively.

**Interpretation:**

While there may be common preconditions at the systems, organisational, professional and patient level, effective MDT-care was likely to be goal and context specific. The introduction of MDTs will require careful planning and implementation to ensure that the potential benefits of MDT are realised and that it does not compromise the quality of primary care.

**Funding:**

The International Centre for Future Health Systems is supported by funding from 10.13039/501100001047The Ian Potter Foundation.

**Research in context**

**Evidence before this study**

Multi-disciplinary team care is being promoted world-wide as part of primary care reforms on the assumption it will improve the quality of care. While these assumptions are widely held, MDT-care is conceptualised and implemented very differently in different contexts. Clear evidence is required to inform primary care reforms as to the benefits associated with MDT-care and how it is best implemented to suit local needs and contexts.

A standardised search strategy was used to identify relevant peer-reviewed studies in PubMed, Embase, CINAHL, PsycINFO, PAIS, Web of Science, and Scopus. The search terms included the different terms for primary care (‘primary care’ OR ‘general practice’ OR ‘primary health care’ OR ‘primary healthcare’ OR ‘GP’ OR ‘general practitioner’ OR ‘family physician’ OR ‘general practice nurse’ OR ‘primary care nurse’), multi-disciplinary team care (‘multi-disciplinary’ OR ‘multidisciplinary’ OR ‘inter-disciplinary’ OR ‘interdisciplinary’ OR ‘inter-professional’ OR ‘interprofessional’ OR ‘collaborative practice’ OR ‘team-based care’ OR ‘teambased care’) and continuity of care (‘continuity of care’ OR ‘care continuity’ OR ‘fragment∗’). Articles extracted were published in English between 1 January 2014 and 13 August 2024 to capture recent developments in primary care reforms. The search was updated for the period 13 August 2024 to 13 August 2025. Articles were included if they reviewed or evaluated models of multi-disciplinary teams (MDT) where they related to introduction of non-general practitioner primary care providers into primary care practices—this includes but was not limited to physician assistants, nurse practitioners, practice nurses, paramedics, non-dispensing chemists, psychologists, physiotherapists, dieticians, health educators, and social workers. Sources were excluded if they related to MDT-care in other settings, such as hospitals.

**Added value of this study**

This review identifies mixed outcomes from MDTs in primary care. The review shows the preconditions for MDT to be effective are not created in policy alone, but by organisations and professions implementing MDT-care.

**Implications of all the available evidence**

Research should examine the best use of MDT-care in primary care, specifically if and how it benefits continuity of care and health outcomes for specific cohorts and reduces pressure on the health system overall. The introduction of MDT-care should be closely monitored to ensure the desired outcomes are achieved.

## Introduction

Health systems world-wide face challenges from growing and ageing populations with increasingly complex health needs. Primary care offers opportunities to address many of these challenges through prevention, early detection and management of disease, reducing overall long-term costs to the health care system.^1^^,^^2^ However, primary care needs to be supported, resourced and strengthened to meet these challenges and deliver high quality health care according to Starfield’s ‘Four C’s’ of first contact (access), comprehensiveness, continuity and coordination.^3^

In Australia, the Strengthening Medicare Taskforce,^4^ convened to address challenges in meeting complex health needs, identified four reforms to increase access to primary care, encourage multidisciplinary team-based (MDT) care, modernise primary care, and support cultural change. Taskforce recommendations on how to implement MDT-care reforms include improving the supply and distribution of the workforce, reviewing barriers and incentives for professions to work to their full scope of practice, and providing investment to support MDTs that reflects local need.^4^ Structural changes are required to fund and deliver more preventative and early intervention services and provide comprehensive care to address unmet and unrecognised need.^4^ Changes must support continuity of care, recognising the benefits of relational, information and managerial continuity of care^5^, ^6^, ^7^, ^8^, ^9^ and patient expectations to see their own General Practitioner (primary care/family physician) when they need to^10^.

MDT-care is considered to be *additive*, differing from interdisciplinary (interactive) and transdisciplinary (holistic) forms of team care.^11^ The use of teams or groups to deliver health care is not new^12^ and is not ubiquitous. In 2020, the Organisation for Economic Cooperation and Development (OECD) reported MDTs were used in less than half of member countries and suggested technology be used to support their uptake.^1^ Their success in hospital settings is no guarantee of their success in primary care. In the UK, there has been criticism of MDTs substituting rather than supporting General Practitioners leading to poorer health outcomes; in Canada, there has been criticism that poor design has led to cost blowouts and maldistribution of services.^13^ Recent reviews have either made brief reference to MDT-care^14^ or focused on elements of MDT, including the role of nurses as care coordinators^15^ and the use of micro-teams, to facilitate continuity of care within large practices.^16^ The reviews fail to explain whether MDTs can strengthen primary care, their impact on quality of care (the four Cs) and specifically continuity of care, or how to implement and benefit from MDTs in primary care settings. Therefore, driven by the recent Australian reforms,^4^ this review asks:

Can the use of multidisciplinary teams strengthen primary care?

i. What is the impact of multidisciplinary team care on quality of care and specifically on continuity of care?
ii. What are the enablers and barriers of implementing multidisciplinary team care?

## Methods

A scoping review was undertaken following Arksey and O’Malley,^17^ following a registered protocol,^18^ and reported using the Preferred Reporting Items for Systematic Reviews and Meta-Analyses Extension for Scoping Reviews (PRISMA-ScR).^19^

### Search strategy and selection criteria

For this study, MDT-care means the incorporation of additional health professionals beyond General Practitioners into primary care practices. Peer-reviewed journal articles published in English between 1 January 2014 and 13 August 2024, and extended to 13 August 2025, that reviewed or evaluated models of MDT were included to capture recent developments in primary care reforms; specifically, original research reporting the form, implementation, and any outcomes reported for staff and patients from MDT-care. Studies about MDT-care in hospitals, or integration of care across general practice and hospitals, were excluded (Appendix p 1).

A standardised search strategy, developed in consultation with all authors to ensure precision, reduce bias, and enhance comprehensiveness, was used to identify relevant studies in PubMed, Embase, CINAHL, PsycINFO, PAIS, Web of Science, and Scopus (Appendix p 2). Searches were conducted by the lead author on 13 August 2024 and repeated on 13 August 2025.

All search results were imported into Covidence software (www.covidence.org) and duplicate records were automatically removed. In both the initial search and the updated search, the same two authors screened the titles and abstracts and performed the full text review. The reason for exclusion at the full-text screening stage was documented and fell into two categories: MDT used in or across other health care settings, and contextual papers that identified the need for MDT-care or related to primary care more broadly. Disagreements were resolved by discussion.

### Data analysis

The Excel data extraction template included author, title, publication details, and abstract. For articles considered in scope, data recorded included the jurisdiction, study objective, method, perspectives included, scope of MDT-model, professionals included, outcomes reported for staff and patients, and enablers and barriers to implementation.

The results were thematically analysed to identify the characteristics of MDT models used and any outcomes reported for patients and staff. This approach suited the heterogeneity of the studies extracted. Given the complexities of interacting, interrelated and interdependent systems, enablers and barriers to the implementation of MDT-care were extracted using a socioecological model.^20^^,^^21^ This identified system, organisational, professional, and patient-level factors impacting the implementation of and outcomes achieved by MDT-care. The results were analysed thematically and grouped by research question and emerging themes.

### Ethics

Ethics approval is not required for this scoping review of published literature.

### Role of funding source

The International Centre for Future Health Systems, where four of the authors are based, is supported by funding from The Ian Potter Foundation. The funder has had no role in the study design; the collection, design and interpretation of data; in the writing of the manuscript; or in the decision to submit this paper for publication.

## Results

**Fig. 1:**
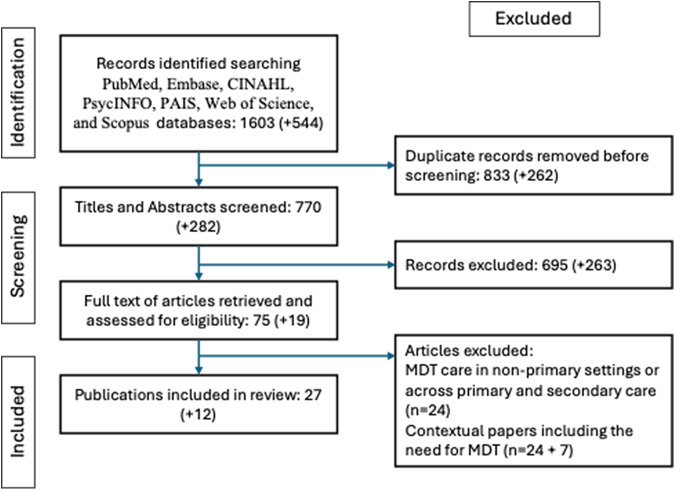
Identification and selection of articles for inclusion in the review (PRISMA flowchart) from January 2024–August 2025– including from references. Note: Additional results from August 2024 to August 2025 search shown as additional results in parentheses.

Our initial search yielded 1603 records and after the removal of duplicates 770 records were screened. Of the 75 full-texts reviewed, 27 met the inclusion criteria. The updated search yielded a further 282 articles after duplicates were removed. Of 19 full-texts reviewed, a further 12 papers met the inclusion criteria which reflects the growing interest in MDT-care as part of primary care reforms (Fig. 1). This additional search highlighted the recent growth in MDT research. In total, 39 studies were in scope for analysis.

A summary of the included articles is available in Table 1.^14^, ^15^, ^16^^,^^22^, ^23^, ^24^, ^25^, ^26^, ^27^, ^28^, ^29^, ^30^, ^31^, ^32^, ^33^, ^34^, ^35^, ^36^, ^37^, ^38^, ^39^, ^40^, ^41^, ^42^, ^43^, ^44^, ^45^, ^46^, ^47^, ^48^, ^49^, ^50^, ^51^, ^52^, ^53^, ^54^, ^55^, ^56^, ^57^ They related to 21 individual countries with some related to specific regions.^22^, ^23^, ^24^, ^25^, ^26^, ^27^, ^28^, ^29^, ^30^, ^31^, ^32^^,^^34^, ^35^, ^36^, ^37^, ^38^, ^39^, ^40^, ^41^, ^42^, ^43^, ^44^, ^45^, ^46^^,^^48^, ^49^, ^50^, ^51^, ^52^, ^53^, ^54^, ^55^, ^56^, ^57^ One provided an international comparison between Ireland and Northern Ireland, and another provided a comparison between Cananda and Norway.^33^^,^^47^ Practice in Canada received the most attention (n = 8) followed by the US (n = 5). Three studies related to systematic or scoping reviews and included multiple countries.^14^, ^15^, ^16^ Studies used a mix of quantitative and qualitative methods; studies examined patients’ potential acceptance or perspectives of MDT-care,^22^^,^^31^^,^^35^^,^^42^^,^^54^^,^^57^ the implementation of MDTs,^28^^,^^32^^,^^33^^,^^50^^,^^52^^,^^56^ and outcomes from MDT-care.^23^^,^^24^^,^^26^^,^^29^^,^^38^^,^^44^ One study focused on financial arrangements.^27^

**Table 1:** Summary of articles determined in scope.

Reference	About the study	Findings (practices/patients)	Enablers and barriers to MDT-care and continuity of care
Bonney et al. (2014)^22^	• Context: Australia • Objective: To explore the conditions under which older patients would accept having someone other than their General Practitioner (GP) involved in their care for chronic conditions. • Method: Survey. • Perspective: Patients' (60 years and older) in 10 practices (n = 272). • Form of MDT care: Hypothetical research on whether MDT-care would be acceptable.	Patients appear to seek continuity of care. Patients were more comfortable with GP than practice nurse. Comfort with practice nurse was positively associated with increased contact with GP, and negatively associated with the number of chronic conditions, and not associated with the frequency of other health professional visits. Need to ensure continuity of care is not lost in health care reform. ‘Continuity of care matters (Guthrie et al., 2008), particularly for older patients and those with chronic conditions (Nutting et al., 2003), as demonstrated in this study’ (p32)	• Enablers: Additionality of services offered—education, measurements, advice, care coordination • Barriers: Not reported.
Wetmore et al. (2014)^23^	• Context: Canada (Ontario) • Objective: To examine patient satisfaction with access and continuity of care in an MDT. • Method: Survey • Perspective: Patients (n = 301, 18 years or older) • Form of MDT care: Family practices include social workers, public health nurses, practice nurses, laboratory services and a shared mental health care program.	Satisfaction associated with access and continuity of care. Satisfaction varied–some were less satisfied with reduced continuity with a GP, others were more satisfied as they felt connected to other members of the team. Continuity of care–no matter who with–appears to be valued by patients and should be a priority for any primary care. Patients were not happy with longer wait times–so reducing wait times was also important.	• Enablers: Support patient contact with usual GP; advanced scheduling systems to increase access. • Barriers: Not reported.
Cueto-Manzano et al. (2017)^24^	• Context: Mexico • Objective: To compare outcomes for GP care and MDT-care for patients with chronic kidney disease. • Method: Prospective cohort study—multiple intervention model v. conventional health model. • Perspective: Patients (n = 39 in each cohort) • Form of MDT care: MDT (comprising GPs, social workers, dieticians, physical trainers) focusing on people with chronic kidney disease. Model also includes self-help groups.	Found better outcomes for some measures (lifestyle factors) than others using MDT-care. This reflects broader scope of other practitioners (relative to GPs). Better results than earlier studies reflect more opportunity through earlier intervention (stage 1 and 2 of disease).	• Enablers: Staff training. • Barriers: Not reported.
Ehman et al. (2017)^25^	• Context: US • Objective: To explore differences in patient preferences for continuity of care for healthy patients versus patients with multimorbidity. • Method: Survey of patient satisfaction. • Perspective: Patients (n = 770) • Form of MDT care: Family medicine clinic includes GPs and nurses, and assisted by pharmacists, social workers, psychologists and behavioural health providers.	Patients prefer continuity of care with their primary care provider for chronic disease management. Patients value quick access to care for acute problems. However, for acute visits, patients with chronic diseases prefer to wait longer to see their normal provider than healthy adults. The study highlights the importance of preserving continuity of care patients living with multimorbidity for both acute and chronic appointments.	• Enablers: Speed of access for acute issues; continuity for chronic patients. • Barriers: Not reported.
Warmels et al. (2017)^26^	• Context: Canada (Ottowa) • Objective: Test shared care between GPs and nurses for ‘well child care’ program (early childhood vaccinations). • Method: Qualitative study (chart audits and interviews) • Perspective: GPs, nurses, and patient’s parents. (chart review n = 20; interviews with parents and nurses for each case, focus group of participating GPs) • Form of MDT care: Alternating appointments between GPs and nurses.	Patients accept shared care knowing the GP is close to hand if there are concerns. Nurses were confident knowing they had the support of physicians if needed. GPs said nurses could spend more time with patients and they may open up more. Also have different skill sets.	• Enablers: Co-location; access to other team members as needed; relational continuity between team member and patient; electronic health records. • Barriers: Alternating visits reduced continuity of care with GPs; complexities of billing.
Wranik et al. (2017)^27^	• Context: Canada (3 provinces) • Objective: To develop a framework for the conceptualisation and analysis of financial arrangements in interdisciplinary primary care teams. • Method: Qualitative study. Interviews, round table and policy document review. • Perspective: Providers (n = 19 representing 2015 clinics in 3 provinces; one research roundtable with 14 decision makers or researchers) • Form of MDT care: Different models in different provinces.	Provides a mechanism to study financial models in interdisciplinary team care. Optimal arrangement is context dependent. However, authors suggest ‘incentives are strongest when provider remuneration is interdependent and combined with a team funding model that is linked to whole team activities’ (p9). Financial hierarchy, both overt and covert, is considered to be a barrier to collaboration	• Enablers: Supportive, clear and transparent processes; institutional reinforcements; collaboration • Barriers: Insufficient education and training; mismanagement of resources; team diversity (creating siloes, imbalanced power relations, unequal pay); miscommunication.
Fernald et al. (2019)^28^	• Context: US (Colorado) • Objective: To identify the transformation process required to implement patient centred medical home in the US (Colorado). • Method: Mixed methods, surveys and interviews. • Perspective: Staff (n = 11 interviews; surveys in 11 practice sites). • Form of MDT care: MDT includes clinicians and medical residents.	Takes time, effort and external support ($, facilitators, learning meetings, evaluation, curriculum guidance, consultation tools and resources) to implement. Barriers to MDT-care remain, including inadequate payment models, inflexible staff roles, and difficulty in accessing clinical data.	• Enablers: Shared leadership; cultural change; quality improvement processes; staff engagement; team-based care; patient access; IT systems; patient engagement; care coordination. • Barriers: Progress not linear; inadequate payment models; inflexible staff roles; difficulty accessing clinical data.
Liang et al. (2019)^29^	• Context: Taiwan • Objective: To estimate the effect of shifting from usual care to integrated care and identifies contextual factors that may distort program implementation. • Method: Quant study 2009–2013. • Perspective: Patients (n = 160,000). • Form of MDT care: Community health care groups which include a blend of specialities. The use of a family doctor plan which targets high cost and chronic patients, incorporating elements of integrated care and MDT.	Context in which reform occurs matters. No improvements to coordination of care and reductions in continuity of care. No impact on avoidable admissions and ED visits. Authors suggest this is because models from high income countries are not directly transferable to low-medium income countries and require more systemic changes to enable these to work.	• Enablers: Context specific enablers. • Barriers: Absence of registration and gatekeeping systems; limited capacities of clinics; lack of service remuneration.
Jimenez-Carrillo et al. (2020)^30^	• Context: El Salvador • Objective: To explore the functioning of a network model of PHC, its management of non-communicable diseases, and the role of social participation to strengthen non-communicable disease services. • Method: Qualitative case study. • Perspective: Patients (n = 14) and providers (n = 12) • Form of MDT care: Health care networks divided into two types of teams to deliver promotion, prevention, diagnosis, treatment, management, rehabilitation and palliative care. Community family health teams (GPs, nurses, health promoters, and polyvalent staff member) and community health teams with specialities (GP, paediatrician, obstetrician and gynaecologists, dentists, educators, psychologists, physio, statisticians and lab technicians).	Introduces two levels of organisation. The model has been positively received, where social engagement and NHF drive accountability and promote culture of health prevention. Need to increase knowledge to address inequalities in health by strengthening PHC and its management of non-communicable diseases.	• Enablers: Governance mechanism (health committees involving staff, coordinators and community representatives); promote the right to health care. • Barriers: Lack of understanding of model by hospital staff meaning patients remain under care of hospital longer than they should; lack of medications and lab tests; lack of public transport to access services; lack of coordination between levels of care (need better referrals and counter referrals); lack of workforce; lack of health promoters.
Ashcroft et al. (2021)^31^	• Context: Canada (Ontario) • Objective: To examine how patients with depression and anxiety perceive quality of care from primary care teams. • Method: Qualitative study (interviews and focus groups). • Perspective: Patients (n = 40). • Form of MDT care: Family health teams are interprofessional teams (GPs, nurses, social workers, counsellors, pharmacists, dieticians and others), focusing on continuous coordinated care.	Team-based primary care appears to be the optimal location for mental health services as it builds on existing relationships. However, variations in MDT furthers inequity. If MDT-care is introduced it needs to be consistent.	• Enablers: Free at point of service; reduced stigma. • Barriers: Lack of MDT staff; lack of information about who patients could access.
Badora-Musiał et al. (2021)^32^	• Context: Poland • Objective: To describe the development and implementation of MDT pilot ‘PHC PLUS’ in primary health care (PHC) pilot to improve preventative health care and health promotion. • Method: Descriptive study. • Perspective: Policy makers. • Form of MDT care: Primary health care team pilots (GPs, nurses, midwives, health educators, dieticians, physios and specialist coordinators)	Highlights the difficulties in implementing MDT-care in Poland from a policy perspective.	• Enablers: Legislation; financial incentives; adaptation • Barriers: Not suited to all practices; workforce shortages
Corry et al. (2021)^33^	• Context: Ireland and Northern Ireland • Objective: To understand the feasibility of anticipatory (rather than unplanned) care planning and develop recommendations for its successful implementation. • Method: Part of larger trans-jurisdictional clustered RCT. • Perspective: Nurses doing home visits who assessed patient health (n = 16) • Form of MDT care: Nurse led person-centred anticipatory care planning for older adults at risk of functional decline.	GPs were thought to provide best anchor for partnership between nurses and GPs, although concerns were raised about the time required.	• Enablers: Use of MDT for right patients; GP buy-in; integrated working; nurse training; health literacy. • Barriers: Time; resources; fragmentation; geographic inequities.
Karam et al. (2021)^15^	• Context: Synthesis • Objective: To synthesise empirical studies of care coordination by nurses for complex patients in primary health care. Specifically, what interventions are currently performed by registered nurses in primary healthcare? Who are the target complex patient populations? What activities do these interventions involve? • Method: Scoping review • Perspective: Mixed • Form of MDT care: Focus on nurses coordinating care—targeting patients, family and care givers; targeting health and social care teams; bringing together patients and professionals.	Highlights importance of care coordination in MDT primary health teams, the importance of relational continuity, and the need for home visits to assess vulnerability/functional decline and what supports exist. Identified three types of care coordination: communication and information transfer; increased intensity and frequency of activities; and importance of relational continuity and home visits. PHC needs MDT to deliver integrated care and deliver intensive and efficient care coordination.	• Enablers: Not reported • Barriers: Not reported
Lukey et al. (2021)^34^	• Context: Canada (four provinces) • Objective: To describe how different policies, contexts and innovations facilitate or limit integrated health systems through integrated PHC teams (BC, Alberta, Ontario and Quebec). • Method: Desk top review and cross case comparison. • Perspective: Government. • Form of MDT care: Various models of integrated primary health care across jurisdictions. Variation within models due to composition of teams. Models include primary care, social services, housing and education to focus on the social determinants of health.	Each province varies and thus provides an opportunity to learn. Analysis focused on five most common themes: patient-centred care, team structures, information systems, financial management, and performance measurement.	• Enablers: Co-creation of structures and processes; single comprehensive electronic health record; funding model to support all staff; measurement framework to drive action and performance; integration at local level; integration at health system level. • Barriers: Financial remuneration to establish team care and to sustain team care; IT; professional organisations. Inconsistent reporting makes it hard to understand impact.
Sanyer et al. (2021)^35^	• Context: US • Objective: To understand the patient perspective of electronic health records in team based care. • Method: Qualitative study (focus groups). • Perspective: Patients (n = 25 patients, including community leaders of ethnic, racial and social minority groups). • Form of MDT: includes GPs, nurses, psychologists, social workers, advocates, wellness coaches and nutritionists.	Participants saw records as important, and were happy to share basic information (but did not define what this included). Patients were concerned about errors and limited options to correct them. Patients wanted to control information recorded and shared.	• Enablers: Electronic health records enable informational continuity of care. • Barriers: Inadequate patient records; lack of trust (and disclosure)
Brooks et al. (2023)^36^	• Context: Canada (Ontario) • Objective: To understand how family health teams in Canada Ontario developed interprofessional chronic disease management programs. This identified successes and areas for improvement. • Method: Single case study of SW Ontario Family Health Team to understand how it developed and implemented specific interprofessional clinics. • Perspective: Health professionals (n = 22). • Form of MDT care: Family health teams are interprofessional teams (GPs, nurses, social workers, counsellors, pharmacists, dieticians and others), focusing on continuous coordinated care.	Specific clinics for chronic illness can have positive (focused, additional care) and negative consequences (siloed, uncoordinated care). ‘The question of which organizational structures are required for a primary care team to sustain effective interprofessional collaboration over time remains unanswered.’ (p7) In terms of implementing the multi-disciplinary clinics, it required both collegiality (to improve communications and shared learnings), AND formal communication and process structures to optimise us of resources and avoid internal fragmentation of care for complex patients.	• Enablers: Team building; collegial learning; sharing expertise; communication; shared record system with inbuilt communication system • Barriers: Fragmentation and siloes created need for care coordination; MDT not co-located
Coombs et al. (2023)^16^	• Context: N/A • Objective: Large primary care clinics threaten the continuity of care. To examines how micro-teams are described and opportunities they provide for practice staff and patients, and limitations to their implementation. • Method: Systematic review • Perspective: Practice, staff and patients • Form of MDT care: Large primary care practices including GPs, nurses, pharmacy, physician associates, occupational therapists, physios, dieticians, health coaches and paramedics.	Use of Micro-teams within large primary care practices can facilitate strong relationships and moderate losses to continuity of care.	• Enablers: Optimal team size to ensure continuity of care (unclear what is optimal). • Barriers: Not reported
Everett et al. (2023)^37^	• Context: US • Objective: To understand the relationship within teams, between teams, and between types of primary care providers and continuity of care. • Method: Cohort study • Perspective: Patients (n = 18,808 patients affiliated with 26 practices) • Form of MDT care: GP, physician assistant and nurse practitioner	Most primary care MDTs aim to minimise patient sharing in to preserve relational continuity of care. This study supports this statement. Bigger teams with more different providers within it led to less continuity of care. Continuity of care is inconsistent within MDT-care and is sensitive to panel size and GP interdependence. Therefore, it is critical to align patients with team structures that can achieve their goals. However, team structures should be avoided if continuity of care is the goal; or supplemental providers should be reduced to one. This provides continuity for those who need it and options for those who do not.	• Enablers: Panel characteristics (interdependence with other doctors/providers and panel size) • Barriers: Continuity of care varies widely within the same clinic so must be monitored. Gender (women saw biggest reduction in continuity of care); multi-morbidity (lower continuity for two conditions but not more).
Goh et al. (2023)^38^	• Context: Singapore • Objective: To examine the quality of care for people with type 2 diabetes through three different models (GP led, group and cluster). • Method: Mixed methods—self reporting of quality of care. • Perspective: Patients (assessments n = 343; interviews n = 24). • Form of MDT care: GPs, nurses and care coordinators. Consider three variations—GP led (clinical and administrative), group primary care network (led by two large GP groups) and clusters (partnerships between single GPs and regional health clusters that include polyclinics).	Model of practice did not seem to affect patient outcomes. Patients received integrated care consistent with chronic care model. They had good continuity of care with their GP which may reflect the size of practice many of the patients attended (many single GP practices). Follow up and coordination needed to be improved. This may reflect coordinators being remote from GPs in many situations.	• Enablers: Financial incentives. • Barriers: Lack of coordination; lack of follow up.
Khatri et al. (2023)^14^	• Context: N/A • Objective: To synthesise evidence on different levels of care coordination of primary health care (PHC) and primary care. • Method: Scoping review • Perspective: Mixed • Form of MDT care: Different themes of care coordination for different stages of conditions (prevention to rehabilitation) across the life-course, across professions and sectors.	Care coordination occurs at individual, organisational and system levels. Factors affecting care coord including patient, providers and departments/sectors. Health systems should focus on care coordination–ensuring care meets health needs at different stages of health conditions by a MDT. Need to think within and beyond the health sector.	• Enablers: Staff training; resources • Barriers: Lack of staff training; lack of resources
Mathew et al. (2023)^39^	• Australia (Queensland) • Context: • Objective: To evaluate a pilot program and consider the process, engagement and quality of care outcomes. • Method: Quantitative case study of service use (process and outcomes evaluation). • Perspective: Practice (up to n = 1606 records). • Form of MDT care: Patient centred medical home model of team based care. Aims to strengthen access, relationships, engagement and agency using an MDT that has an expanded and intersection scope of practice.	Team seems to be getting more complete information from patients to determine risk of cardio-vascular disease. Continuity is similar for those who enrol in MDT-care and those who do not. Patients prefer to see an Aboriginal Health Worker. Unclear whether MDT provides choice or improved coordination.	• Enablers: Not reported • Barriers: Not reported
Næss et al. (2023)^40^	• Context: Norway • Objective: To identify the preconditions for proactive and interdisciplinary follow up of older patients receiving home health care. The aim is to shift from reactive to proactive healthcare with early identification of symptoms and functional decline of older people. • Method: Qualitative study focus group interviews • Perspective: Providers (n = 41 from across 3 districts) • Form of MDT care: GPs working with nurses to deliver home health care.	Screening needs to be backed up by planning and leadership.	• Enablers: Common goals; sharing tasks; common understanding of each other’s professions; clinical leadership; promotion of integrative and proactive care; interdisciplinary coordination. • Barriers: Professional and administrative challenges; GPs and nurses need to understand each others role and responsibilities; inadequate patient records.
Chance-Larsen et al. (2024)^41^	• Context: Norway • Objective: To examine attitudes to GPs and physios working together to enable task shifting of musculoskeletal health work from GPs to physios and what is required to support this change and develop MDT working. • Method: Qualitative study (interviews) • Perspective: GPs (n = 5) and physios (n = 11). • Form of MDT care: Task shifting from GP to physio.	Task shifting rather than providing additional services. Both competition and cooperation exist between GPs and physiotherapists around management of musculoskeletal disorders in Norwegian primary care.	• Enablers: Investment in life-long training. • Barriers: Professional boundaries; regulations; financial barriers; lack of mandatory training; lack of skills/experience to identify red flags; potential to reduce competency when diverting care to others; professional organisations can create barriers to implementation.
Donaghy et al. (2024)^42^	• Context: Scotland • Objective: To explore patients views on changes to general practice in Scotland since expansion of MDT to enable GPs to spend more time as expert medical generalists with patients with complex needs. • Method: Qualitative study of patients from different socio-economic areas and rural/remote areas. • Perspective: Patients (n = 30, split across each area) • Form of MDT care: MDT with GPs and other primary care providers (unspecified).	While positive about MDT staff, patients still wanted to see a known GP for health concerns they considered potentially serious. This was more of a concern for patients with co-morbidity and from deprived areas.	• Enablers: Enabling environment through policy and payment reform. • Barriers: Needing to share private information with reception to be directed to appropriate team member.
Floriancic et al. (2024)^43^	• Context: Canada (Ontario) • Objective: To explore chronic disease management practices implemented by nurse practitioners within Nurse Practitioner Lead Clinics in Ontario • Method: Qualitative (interviews) • Perspective: Nurse practitioners (n = 11) • Form of MDT care: Nurse practitioner led clinics, staffed by registered practice nurses, registered nurses, consulting physicians, dieticians, pharmacists, physiotherapists, social workers and community health workers	Nurse Practitioner Clinics is one of several models of primary care in Ontario. The nurse led interprofessional team approach provides comprehensive care to clients with complex presentations who have trouble registering with a GP clinic. Main themes identified were: (1) bridging access to clients who fall between the cracks, (2) benefits of interprofessional care, (3) underappreciation of nurse practitioner knowledge and skill level, and (4) addressing healthcare system burden.	• Enablers: Less power differential between providers enabled greater collaboration and development of care plans. Nurses led a holistic approach to care. • Barriers: Lack of understanding of scope of practice (believed limited ability to order tests, prescribe medications or make specialist referrals).
Goff et al. (2024)^44^	• Context: United Kingdom • Objective: To understand the impact of primary care networks (PCNs) (groups of practices) on continuity of care. • Method: Qualitative study in 19 practices in five primary health networks. • Perspective: Health care professionals (n = 33) and patients (n = 35; over 65s with polypharmacy, with anxiety or depression, or 18–45). • Form of MDT care: includes GPs, nurses, pharmacists, physician associates, healthy ageing coordinators, patient ambassadors, and mental health staff. Model diverts patients with minor illnesses to non-GPs. PCNs also provide hubs and clinics for chronic conditions.	Larger scale required better care coordination and information sharing processes to improve care for vulnerable or priority groups. The new arrangement undermined continuity of care. Continuity of care understood as relational continuity by participants.	• Enablers: Reimbursement scheme for additional roles; emphasis on documentation and shared patient records with option to flag and escalate/de-escalate/task requests between providers; care coordinators • Barriers: Creates more fragmentation so needed to add care coordinators; prioritised continuity for some patients over others; systems lacked interoperability.
Huyen et al. (2024)^45^	• Context: Vietnam • Objective: To describe how teams collaborates. Provides perceptions and experiences of primary health care providers about interprofessional collaboration. • Method: Qualitative study (focus groups and interviews). • Perspective: Providers (different professions). Focus groups (n = 2) and interviews (n = 15) • Form of MDT care: Community health centre comprising GPs, physician assistants, nurses, pharmacists, midwives, and traditional physicians.	Not all sites had all disciplines for MDT-care in place. Where in place, requires education program and training to address deficiencies in three areas: Lack of collaborative practice, knowledge, and facilitators and barriers to collaboration. Lack of shared decision making in patient centred care.	• Enablers: GP-led care. • Barriers: Lack of staffing across disciplines; lack of collaborative practice (communications, teamwork, roles and responsibilities); conflict within team concerning others lack of action; hierarchies; unclear role of pharmacist.
Kayira et al. (2024)^46^	• Context: England • Objective: To understand the impact of practice list size and workforce composition (grouped into small GP reliant practices, medium sized GP-led with MDT input and large MDT practices) on clinical outcomes and patient experience. • Method: Quantitative, using practice data from 6024 practices (93% population) • Perspective: Practice • Form of MDT care: Primary care networks (GPs, nurses, pharmacists, health care assistants, admin, allied health) who also work closely with other services including social care and community services.	Some aspects of care better at a small scale (access, continuity of care, experience and satisfaction), some at larger scale (screening). Small practices had higher patient reported indicators on all but ‘confidence and trust’ where medium practices did better. Large practices had higher cancer detection rates.	• Enablers: Not reported • Barriers: Not reported
Nystrøm et al. (2024)^47^	• Context: Canada and Norway • Objective: To explore patient pathways in primary healthcare from different health personnel’s perspectives from primary care organisations in Canada and Norway • Method: Qualitative interviews • Perspective: Physicians, nurses and managers (n = 19) • Form of MDT care: Physicians, practice nurses, registered nurses, podiatrists, pharmacists, obstetricians and gynaecologists	While the settings were different, both had specialised and centralised programs in prevention, education, and chronic disease management. Participants expressed concerns related to health care services being organized in different levels, and an increasing specialization of primary care rather than comprehensive care. As a result it was challenging to achieve coordination or continuity. More integrated care initiatives should be prioritized in the development of primary care. This includes complementary work at the micro, meso and macro level: i.e., at the clinician level, at the organizational level, and at the system level.	• Enablers: Integration strategies to reduce siloed thinking and fragmentation; dedicated physician to ensure continuity of care and be engaged in what the team is doing for the patient; electronic health records to support the coordination and sharing of information across the team. • Barriers: Structural challenges; shift to specialised primary care; differences in health care levels, funding systems, management, electronic health records, and organisations; high staff turnover challenged the continuity of care.
Wells et al. (2024)^48^	• Context: New Zealand (North Island) • Objective: To explore the views of general practitioners, nurse prescribers and pharmacist prescribers about their role in managing medicines-related continuity of care. • Method: Qualitative (interviews) • Perspective: Prescribers (n = 16, from 8 practices) • Form of MDT care: Focused on GPs, nurse prescribers and pharmacist prescribers withing broader MDT	Prescribers identified the important connection between continuity of care and achieving good outcomes from medicines. Good patient-prescriber relationships and ongoing interdisciplinary relationships across all health settings were considered essential to medicines-related continuity of care. Prescribers experienced challenges associated with increasing multimorbidity, medicines complexities, and fragmentation of clinical records. With a range of health disciplines now prescribing medicines concurrently in general practice, more effort was needed to understand how prescribers with various scopes of practice could work collaboratively to support safe, effective and equitable outcomes from medicines. Greater emphasis was required to develop good relationships with patients, improve interdisciplinary working relationships, and have consistent and accurate exchange of medicines-related information, within and across all health settings. Ultimately, GPs were seen as responsible for ensuring patients’ entire medicine regimen is safe and effective when more than one prescriber was involved.	• Enablers: Continuity of care; good patient-prescriber relationships; interdisciplinary relationships across all health settings; accurate information exchange within and across health settings. • Barriers: Increasing multimorbidity; medicine complexities; fragmentation of clinical records; lack of collaboration across prescribers.
Akbar et al. (2025)^49^	• Context: Indonesia • Objective: To explore healthcare providers' interprofessional collaboration experiences with the integrated information system for non-communicable disease management in primary care • Method: Focus groups • Perspective: Providers (n = 15) • Form of MDT care: Teams focused on non-communicable disease comprising nurses, GPs, midwives, public health workers	Technology supported collaboration, coordination and communication in primary care, and led to comprehensive clinical decision making. The technology optimised patient management by providing accurate and updated patient data, provided a way to monitor patient service progress, and provided an integrated medical record. It also improved health worker accessibility.	• Enablers: Technology enabled MDT workflow, collaboration, communication, and improved access to patient data. • Barriers: Not reported
Alodhialah et al. (2025)^50^	• Context: Saudi Arabia • Objective: To evaluate the implementation of person centred care strategies and understand their impact on health outcomes for older adults in family medicine settings • Method: Mixed-methods (Surveys and interviews) • Perspective: Healthcare providers (survey n = 47, interviews n = 12) and patients 65+ with at least one chronic condition (survey n = 126, interviews n = 11) • Form of MDT care: Team developed person-centred comprehensive care plan for this group; comprising physicians, nurses, pharmacists, social workers and others.	Staff thought both continuity of care, education strategies, and collaboration across an MDT were important in the delivery of comprehensive patient centred care. Patients were very satisfied with the continuity and comprehensiveness of care they received, the communication of care plans, and involvement in decision making about their care (ranging from 75 to 79%). Patients were also satisfied with MDT care (70%). The group had lower hospital readmission rates and this approach to care planning within an MDT was considered to be effective at managing chronic disease and improving quality of life.	• Enablers: Clear communication; coordination; leadership; patient involvement and empowerment; and training in person-centred care and MDT care. • Barriers: Time constraints; workloads; resources; support and infrastructure; insufficient training in person-centred care; patient resistance to change; communication barriers (patient language and literacy)
Darling et al. (2025)^51^	• Context: Canada (Ontario) • Objective: To investigate perceptions of how integrating midwives into primary health care teams impacts access to care • Method: Qualitative • Perspective: Providers and policy makers (n = 28) • Form of MDT care: Integrating midwives into primary care MDTs	Integrating midwives increased visibility and trust of the profession (approachability and acceptability), decreased access barriers such as travel time and cost (affordability), increased collaboration between healthcare providers (appropriateness), and ensured more timely and available care (availability and accommodation).	• Enablers: Accommodation; location; staffing levels; flexibility in when and where care was provided; increased visibility of midwives; removing access barriers; co-location with familiar professions; interpersonal collaboration. • Barriers: None reported
Fumagalli et al. (2025)^52^	• Context: Brazil • Objective: To understand how interprofessional collaborative practices are built in the work process within Family Health Units • Method: Mixed-methods (program evaluation data and interviews) • Perspective: Nurses (n = 14), Community Health Agents (n = 2), Physician (n = 1) • Form of MDT care: Family health units included nurses, community health agents, physicians, dentist, physical therapists	Identified the enablers and barriers to collaboration. Enablers included the willingness to work together, mutual respect, team meetings, activity planning, meetings to discuss cases as well as process issues, having collective spaces, shared/joint patient consultations, group work, clear task allocation, stability in staffing (led to trust and better collaboration), communication (medical records, messaging, team meetings), support for each other (teamwork, unity), support from others (including clinical supervision/mental health supports for workers). Collaborative practices were characterized by actions developed by different professionals with different levels of integration. The study also highlighted the fragmentation of health care and the failure to include the patient in decision-making about their own care.	• Enablers: Collaboration between colleagues—both willingness to collaborate and support each other, and processes and physical spaces that enabled collaboration. Stability in staffing led to better collaboration and trust. Records and systems that enabled sharing of patient information and actions. Clinical supervision and mental health support for staff. • Barriers: Lack of knowledge of integration, high staff turnover, lack of human resources, different working hours.
Groot et al. (2025)^53^	• Context: The Netherlands • Objective: To explore perspectives of GPs, older patients, practice nurses, and assistants, on improving personal continuity in general practice, and to identify barriers and facilitators that affect this improvement process • Method: Qualitative study • Perspective: GPs (n = 17), practice assistants (n = 4), nurses (n = 2), patients (n = 7) • Form of MDT care: GPs, nurses, practice assistants	Personal continuity was considered to be something provided by the entire practice team and not just the GP. GPs were concerned about lost contact with patients and not being able to notice things in patients they did not know as well as a result of MDT care. But GPs also said that some roles suited some tasks better–such as nurses caring for vulnerable older patients. Participants highlighted that good triage processes enabled continuity of care for non-urgent visits. Self-booking systems also allowed patients to exercise choice and self-triage. Retaining continuity of care was particularly important for older frail patients. Practices needed to support the team-based provision of continuous care.	• Enablers: Communication; team stability; use of electronic health records; patient trust in model and providers; access to preferred GP. Smaller teams enabled continuity. • Barriers: Lack of time (to do briefings); patients’ lack of understanding of what different providers do; GPs not being able to step back; insufficient staffing; insufficient management; organisational (staff shortages); and societal barriers (payment system).
Hooberman et al. (2025)^54^	• Context: USA (Michigan) • Objective: To improve multidisciplinary communication, care coordination, and patient satisfaction in primary care. • Method: Pre/Post survey • Perspective: Providers (n = 17), complex patients (n = 31) • Form of MDT care: Program targeted complex patients; provided by GPs, social workers, care navigators, nurses	Complex patients require multidisciplinary input for optimal care, but this can lead to fragmented care. This quality improvement initiative aimed to improve communication, care coordination and patient satisfaction. Team meetings improved communication and patient care. However, there were challenges in selecting patients to participate in the program and the team meeting participants.	• Enablers: None reported • Barriers: Lack of clarity of roles; mode and high frequency of communication impacted collaboration, professional connections and stress levels.
Imison (2025)^55^	• Context: England • Objective: To examine the impact of key roles and skill mix on GP workload, satisfaction, clinical quality and costs, to understand how general practices and primary care networks can realise the benefits of expanded MDTs. • Method: Mixed-methods • Perspective: Providers, patients • Form of MDT care: GPs, paramedics, physician associates, nurses, HCA, NA, pharmacists, pharmacy technicians, pharmacy assistants/dispensers, advanced pharmacy practitioners, paramedics, first contact physiotherapists, care coordinators and social prescribers	GPs make up a decreasing proportion of the general practice workforce in England (now about 40%). The *Additional Roles Reimbursement Scheme* enabled 17 new roles to be included in primary care practices, including clinicians and other staff with direct patient contact, aiming to ensure patients get an appointment with the right professional depending on their needs. In principle, if unable to recruit more physicians, a skill mix was a way to fill the workforce gap. In delivery, practices found it difficult to match patients’ needs to different practitioners. The study also highlighted that some staff with the same job title had very different skill sets (training and experience) leaving some patients requiring follow-up appointments with other practitioners due to unmet needs. GPs were now supervising or supporting colleagues training and supervision–and only seeing patients with complex needs. Patients wanted better information about the roles and skills of other providers. Practices with a higher proportion of GPs had reduced waiting times, and higher patient and GP satisfaction scores. Other professionals had lower patient satisfaction scores.	• Enablers: Time for GPs to supervise and advise other staff; professional standards; training programs; continuity of care prioritised for patients with chronic conditions; shared medical records; clear communication of roles; clinical supervision; training of receptionists to direct patients to rights staff; organisational development; match skills in team to population. • Barriers: None reported
Jokelin et al. (2025)^56^	• Context: Finland (Espoo) • Objective: To evaluate the implementation of a new MDT from 2021 to 2023 to understand its impact on access and continuity of care compared to an established model • Method: Quasi-experimental, comparing 5 intervention centres with 3 control centres. Access and continuity of care measured by access to physician. • Perspective: Patient data. • Form of MDT care: Nurses and physicians.	The new model removes the triage process and brings focus to patient care forward—shifting from managing queues to managing patients. This led to enhanced access but had mixed results in terms of continuity of care.	• Enablers: Open access scheduling, multi-professional consultations (at the same time), physician triage, lean processes • Barriers: None reported
Schütz-Leuthold et al. (2025)^57^	• Context: Switzerland (Vaud) • Objective: To assess patients’ experiences with new nursing activities in general practice • Method: Mixed methods • Perspective: Patient pre-post survey of experience (n = 109), and interview with patients (n = 10) • Form of MDT care: Introduction of nurses to general practice—physicians, nurses	Nurses were introduced to general practice to provide case management–nurses provided follow up care with an individualised care plan. Nurses provided care to patients with chronic illness–in particular care coordination, therapeutic education, and preventive care. The study highlights the potential of nurse-led case management to address gaps in primary care delivery, particularly in managing chronic diseases. The integration of nurses into general practice settings improved the provision of preventive care, enhanced patient education, and increased accessibility to care. Qualitative data supported these results, highlighting the importance of nurses’ accessibility and availability and the holistic nursing care provided.	• Enablers: Information sharing, direct communication between physicians and nurses • Barriers: None reported.

GP—General Practitioner, MDT—Multi-disciplinary team.

### Characteristics of MDT-care

The models of MDT-care reported in the literature varied substantially (Table 1). Models included multiple providers *working together* to care for a patient,^27^ particularly for complex patients^54^; interprofessional teams providing patients the option to see an *alternative provider* of care within the practice^31^^,^^44^^,^^55^; *incorporating different professions* into primary care such as nursing^57^ or midwifery^51^; models where tasks were *shifted* from General Practitioners to alternative providers such as physiotherapists^41^; and models where patients saw *different health practitioners* each visit for the same issue.^26^ Some models were *nurse-led*,^15^^,^^33^^,^^43^ others *General Practitioner-led*^29^; some related to General Practitioners and nurses,^37^^,^^38^^,^^40^^,^^53^^,^^56^^,^^57^ others included a wider range of primary health providers^16^^,^^23^^,^^28^^,^^30^, ^31^, ^32^^,^^42^, ^43^, ^44^, ^45^^,^^47^, ^48^, ^49^, ^50^, ^51^, ^52^^,^^54^^,^^55^; and some included health *and* social services.^24^^,^^25^^,^^34^^,^^36^^,^^39^^,^^46^

MDTs provided care coordination for different conditions across the life-course such as early years or end-of-life care.^14^ MDTs also targeted chronic kidney disease,^24^ diabetes,^38^ other chronic conditions,^36^^,^^38^^,^^43^^,^^44^^,^^50^^,^^57^ health promotion/prevention,^30^ and culturally responsive care.^39^

The disciplines making up MDTs varied significantly by model, but also within models recognising geographical differences in need and context, staffing issues, and practice size.^31^^,^^32^^,^^45^ Such differences could lead to inequities where there were variances across regions.^33^

### What outcomes have been observed?

Reported outcomes from MDT were mixed (Table 2). Positive outcomes were reported when MDTs targeted chronic illness—i.e., where MDTs provided additional care.^31^^,^^36^^,^^50^ Better outcomes were noted for some measures—associated with lifestyle education, early intervention, and collecting complete information from patients—over other factors; this may reflect the additional educational element provided by other health practitioners.^22^^,^^24^^,^^39^^,^^47^^,^^50^^,^^57^ Patients also valued quicker access to care available through some MDT-care models.^23^^,^^25^^,^^43^

**Table 2:** Summary of outcomes.

Positive outcomes	Negative outcomes
• Additional services including education, prevention, screening, and case management.^24^^,^^31^^,^^36^^,^^39^^,^^46^^,^^50^^,^^51^^,^^53^^,^^57^ • Quicker access.^23^^,^^25^^,^^43^ • Care teams specific to chronic conditions^31^^,^^36^^,^^50^ or complex patients^54^	• Greater fragmentation or siloes of care that then required coordination^36^^,^^47^^,^^55^ • Reduced continuity of care^29^^,^^37^^,^^47^ • Privacy concerns^35^ • Different skill levels of staff with same job title leading to patients being referred to other providers^55^

Some studies reported no effect on patient outcomes,^38^ including no improvements to coordination of care, emergency department presentations, or hospital admissions.^29^ Other studies reported negative outcomes explained by MDTs creating siloes of care that required additional coordination,^36^^,^^47^^,^^55^ reducing continuity of care,^29^^,^^37^^,^^47^ or creating concerns about privacy when sharing information across the team.^35^ One study also identified that not all staff with the same job title have the same skills and scope of practice leading patients to need to be referred back to other team members.^55^

Several studies highlighted the importance patients and providers attached to continuity of care, whether that was relational (identified by patients^15^^,^^22^^,^^23^^,^^26^^,^^37^^,^^44^^,^^55^) or informational (identified by practitioners^35^^,^^49^). Some patients reported relational continuity could be established with other providers, such as nurses, although continuity of care with their General Practitioner was preferred.^15^^,^^22^^,^^25^^,^^44^^,^^47^, ^48^, ^49^^,^^52^^,^^57^ Team size was found to be inversely related to continuity of care—the smaller the team, the greater the continuity of care^16^^,^^37^^,^^46^^,^^53^—to the extent that if continuity of care is the priority, it may be argued that ‘supplemental providers should be reduced to one’.^37^ However, larger teams provided other benefits such as higher screening rates.^46^ Evidence was inconclusive about the ideal organisational structure, composition, leadership, or team size required to achieve optimal outcomes.^16^^,^^33^^,^^36^^,^^37^

### Enablers and barriers to implementing MDT-care

Most studies identified enablers and barriers to MDT-care, informing the preconditions required for reform (Table 3). Using a social ecological model, these were identified in terms of systems, organisational, professional and patient level factors that affect the success of MDT-care in general practice.^20^^,^^21^

**Table 3:** Summary of enablers and barriers to MDT-care in general practice.

	Enablers	Barriers
Systems level	• Professional boundaries, regulations and standards^41^^,^^55^ • Establishing policy, legislative and funding mechanisms, including measurement framework, to incentivise MDT-care and create common goals^14^^,^^32^^,^^34^^,^^38^^,^^40^^,^^42^^,^^44^ • Integration strategies to reduce siloed thinking and fragmentation^47^ • Training programs^55^	• Regulatory and financial barriers^14^^,^^28^, ^29^, ^30^^,^^33^^,^^34^^,^^41^^,^^47^^,^^53^ • Lack of mandatory training^41^ • Differences in health care levels^47^ • Shift to specialised primary care (disease focused) rather than comprehensive primary care^47^ • Broader contextual factors (such as lack of transport, medications, laboratory resources)^29^^,^^30^
Organisational level	• Resources to implement the model,^14^^,^^34^ and then continually improving the model, adapting the model to meet the needs of the community and staff^28^^,^^32^^,^^34^ • Organisational, clinical leadership/supervision and professional support and development^28^^,^^33^^,^^40^^,^^50^^,^^52^^,^^55^ • Supportive, clear and transparent processes,^27^ including clinician led triaging^56^ • Sufficient staffing levels^51^ to match the population and their needs^55^ • Team building, training, learning, cultural change, sharing expertise to develop integrated and collaborative working^14^^,^^24^^,^^27^^,^^28^^,^^33^^,^^36^^,^^40^, ^41^, ^42^ • Clear communication of roles^55^ • Improving communication and coordination between team members^36^^,^^50^^,^^53^ • Shared electronic health record,^26^^,^^28^^,^^34^, ^35^, ^36^^,^^44^^,^^47^^,^^49^^,^^52^^,^^53^^,^^55^ with inbuilt communication^36^^,^^47^^,^^49^^,^^52^ to escalate or de-escalate care between team members^44^ • Technology enabled workflow^49^ • Co-location of teams at the same practice^26^^,^^36^ • Optimal team size,^16^^,^^37^^,^^46^ with smaller teams enabling continuity^53^ • Stability in teams and staffing^52^^,^^53^ • Processes and physical spaces that enable collaboration^52^ • Continuity of care for people with chronic or complex conditions^55^ • Training of non-clinical staff to support the model^55^	• Workforce shortages^30^, ^31^, ^32^^,^^45^^,^^50^^,^^53^ • High staff turnover^47^ • Different working hours^52^ • Not suitable to all practices^32^ • Lack of IT systems to support sharing of clinical information^28^^,^^34^^,^^35^^,^^40^^,^^47^ and inconsistent reporting^34^ • Complexities of billing^26^ • Insufficient management, support or infrastructure,^47^^,^^50^^,^^53^ including lack of accommodation^51^ • Location^51^
Professional level	• Role clarity^54^ • Training in person-centred care and. MDT care^50^ • GP led,^45^^,^^47^ with time allocated to supervise and advise other staff^55^ • Improving collaboration and communication/information sharing between professionals who may or may not be co-located,^48^, ^49^, ^50^, ^51^, ^52^^,^^57^ balancing between too little communication and too much communication (which could affect collaboration and stress)^54^ • Improving access to patient data^49^ • Improving care coordination and follow up^28^^,^^38^^,^^40^^,^^44^ • Taking a holistic and comprehensive approach^43^	• Lack of collaborative practice due to professional boundaries, power differentials, unclear roles and responsibilities, and professional organisations forming barriers^28^^,^^30^^,^^34^^,^^40^^,^^41^^,^^43^^,^^45^ • Lack of training and skills, particularly in identifying red flags^41^; potential reductions in competency when diverting care to others^27^^,^^41^ • High workloads/lack of time^50^^,^^53^
Patients	• Making MDT-care attractive to patients by for example providing free services at point of care^31^; faster access for acute patients^25^; advanced scheduling system to increase access and continuity,^23^ or additional services offered (such as education, advice, measurements and care coordination)^22^ • Flexibility in when and where care is available from^51^ and who^56^—with patients having the choice • Co-location with familiar professions^51^ • Involving and empowering patients in MDT care^50^ • Providing MDT-care tailored to specific cohorts/patients needs^33^ • Establishing trust in the model and providers through clear communication^53^ • Providing access to preferred GP^53^ • Strong relationship between patient and provider^48^	• Lack of information for patients and other services to understand the scope of practice for different team members, overlap (or gaps) between scopes of practice, or how to access their preferred provider^30^^,^^31^^,^^43^^,^^48^^,^^53^^,^^54^ • Fragmentation, poor collaboration between providers, or siloes of care^27^^,^^36^^,^^44^^,^^48^ • Potential risk to patients, such as missing red flags^41^ or through miscommunication^27^ • Privacy and trust concerns^35^^,^^42^ • Resistance to change^50^ • Communication barriers (language, literacy, health literacy)^50^

At a systems level, the results showed MDT required clear policy, legislative and funding mechanisms, including a measurement framework, to drive and facilitate implementation.^14^^,^^32^^,^^34^^,^^38^^,^^40^^,^^42^^,^^44^ Integration strategies to reduce siloed thinking and fragmentation also had the potential to create an enabling environment and common goals for practices to work towards.^47^ Other system level enablers included regulations and standards to clarify the scope of different professions,^41^^,^^55^ and training programs that increased the multi-disciplinary and transdisciplinary capabilities of different health professions.^41^^,^^55^ Regulatory and financial barriers were considered key impediments to implementing MDT-care.^14^^,^^28^, ^29^, ^30^^,^^33^^,^^34^^,^^41^^,^^47^^,^^53^ There were also concerns that MDT-care forced primary care towards specialist or disease specific care and away from comprehensive primary care.^47^ In emerging economies, such as El Salvador, other factors impacted access to MDT-care and health services more broadly, such as shortages of public transport, medications, and laboratory resources.^30^

At an organisational level, MDT required resources to implement and to continually improve the model to ensure it continued to meet the needs of the community and staff.^14^^,^^28^^,^^32^^,^^34^ This was in addition to the ongoing cost of delivering MDT-care. Resources alone were insufficient; implementation required organisational and clinical leadership, professional support,^28^^,^^33^^,^^40^^,^^50^^,^^52^^,^^55^ and clear and transparent processes^27^—including clinician led triaging.^56^ Further, when incorporating new professions and new ways of working, efforts were required to support the organisational and cultural change through team building, training and sharing expertise to support collaborative working.^14^^,^^24^^,^^27^^,^^28^^,^^33^^,^^36^^,^^40^, ^41^, ^42^ This worked best when underpinned by shared health records with associated mechanisms for inter-team communication to ensure informational continuity and risks were managed.^26^^,^^28^^,^^34^, ^35^, ^36^^,^^44^^,^^47^^,^^49^^,^^52^^,^^53^^,^^55^ The use of technology also supported workflow.^49^ Two studies flagged the benefits of co-locating teams; this reduced impacts on patients and allowed staff to check with colleagues about a patient’s care as needed.^26^^,^^36^ Other studies identified the need for stability in teams and staffing,^52^^,^^53^ the need for both processes and physical spaces that provided access and enabled collaboration,^52^ and the need to meet patients’ needs while minimising team sizes to ensure relational continuity.^16^^,^^37^ Organisational barriers include workforce shortages,^30^, ^31^, ^32^^,^^45^^,^^50^^,^^53^ high staff turnover,^47^ lack of support or infrastructure,^47^^,^^50^^,^^51^^,^^53^ and the complexities of billing.^26^

At a professional level, MDTs provided opportunities to improve care coordination and patient follow up,^28^^,^^38^^,^^40^^,^^44^ provided there was role clarity^54^ and sufficient clinical supervision.^55^ However, significant barriers were also identified, highlighting conflicting views on professional scope and boundaries, lack of clarity in roles and responsibilities within MDTs, and professional organisations being unsupportive in implementing MDTs in primary care.^28^^,^^30^^,^^34^^,^^40^^,^^41^^,^^45^ One way this barrier may be addressed is by embedding MDT-care in training programs across different professionals.^14^^,^^24^ Another concern at a professional level was the potential reduction not just in continuity of care with a patient, but also reductions in competency when diverting tasks to others^41^; MDTs created tensions between professions through siloed working, imbalances in power, and unequal pay^27^^,^^28^^,^^30^^,^^34^^,^^40^^,^^41^^,^^43^^,^^45^—also discussed in the broader primary care literature concerning interprofessional collaboration.^58^

Patients required a value proposition to make MDT-care attractive. The review included examples of fee-free services at point of care^31^; faster access to care for acute patients^25^; the use of advanced scheduling systems to increase access and continuity^23^; and services that offered additional value beyond seeing a General Practitioner alone (such as education, advice, measurements and care coordination)^22^ including services for specific chronic conditions.^33^ For patients to benefit from MDT they required information about the service and how to use it (including the choices they could make),^30^^,^^31^ and about how information about them would be shared and risks managed.^35^^,^^41^^,^^42^ Patients ultimately valued both timely access to care and continuity of care (specifically relational continuity^48^^,^^53^); this was potentially undermined when MDT led to greater fragmentation.^27^^,^^36^^,^^44^

## Discussion

Policy makers, funders and practice owners lack clear evidence about how MDTs can improve the quality of primary care. This review consolidates the growing evidence of the use of MDTs in primary care to inform policy and practice.^3^ The review identified significant variation in the design and implementation of MDT-care and also highlighted mixed outcomes often driven by different design choices in different primary care contexts. Further, the review showed the preconditions required for MDTs to strengthen primary care are not created in policy alone, but by organisations and professions implementing MDT-care. The growing interest in MDTs in primary care reforms, evidenced by the growth in the literature even in the last year, may be driven by multiple factors, including: increasing pressures on public finances, ageing and growing populations with increasingly complex health needs, and workforce shortages. Despite these pressures, any response must be mindful of the basic principles underpinning effective and accessible health care–Starfield’s ‘Four C’s’ of first contact (access), comprehensiveness, continuity and coordination.^3^ While the research question and analysis focused on continuity of care due to the potential impact MDT may have on relational continuity, we discuss these findings around each of Starfield’s Four C’s.

In terms of *first contact*, there was some evidence that MDTs increased access to primary care in contexts where it was difficult to access a General Practitioner with lower wait times for alternative providers; however, this led to reductions in continuity of care and patients with chronic conditions often preferred to wait to see their regular General Practitioner for acute visits or for issues of concern.^23^^,^^25^^,^^42^ In primary care systems that funded preventative care,^40^ larger MDTs were able to increase access to preventative care through strategic preventative programs, achieving higher rates of cancer screening than smaller MDTs.^46^ Larger teams were also shown to be able to collect more complete information from patients to facilitate screening.^39^

This review also identified how MDTs in general practice can improve *comprehensiveness of care* in some applications. Consistent with the literature,^12^ MDTs that targeted specific chronic conditions resulted in improved patient care relative to patients not accessing MDT-care for the specific condition.^24^^,^^31^^,^^36^^,^^39^ In these examples, MDTs provided additionality to General Practitioner care; other professions provided education, monitoring, service navigation, and care coordination to prevent or reduce the impact of chronic health conditions leading to better health outcomes and greater patient satisfaction.^24^ However, there were some concerns that this led to specialised primary care (disease focused) rather than comprehensive care.^47^ Some MDTs, involving nurses or social/community services, incorporated patients’ social determinants of health, adjusting services to a patient’s living context and supports, and engaging with family and carers, and were therefore able to provide more comprehensive and holistic care.^31^^,^^33^^,^^34^^,^^46^

Personal *continuity of care* over the long-term may include relational, informational and managerial continuity.^5^^,^^6^ The broader literature continues to highlight the value patients place on relational continuity over structure and function.^59^ Yet, MDT-care may be inconsistent with relational continuity when transferring care to other health professionals working with varying levels of collaboration and coordination.^37^^,^^41^ The evidence in this review showed MDT-care reduced relational continuity when models offered alternative care pathways or where patients were triaged to different professions or practitioners depending on need or urgency of need. Relational continuity decreased with increasing team size; i.e., the larger the team, the lower the continuity of care, which goes counter to the recommendations for bigger teams to include multiple disciplines.^13^ MDTs better supported continuity of care in smaller practices^38^^,^^46^^,^^53^ or when using smaller teams where the lead carer (often the General Practitioner or practice nurse) was well-defined and the patient was receptive to this model. While MDT-care sometimes reduced continuity of care with the General Practitioner, patients often had continuity of care with another member of the team such as the nurse or care coordinator.^23^ Whether MDTs support or undermine relational continuity, and how this relates to clinical outcomes, depends on how MDT-care is implemented and supported (such as through informational continuity) and what aspect of continuity is the focus (relational, informational, or managerial^5^^,^^6^). Service providers recognised informational continuity—through electronic health records and clear communication pathways within MDT members—was essential to effective MDT-care record.^26^^,^^28^^,^^34^, ^35^, ^36^^,^^44^^,^^47^^,^^49^^,^^52^^,^^53^^,^^55^ MDT-care has the potential to support or undermine continuity of care, varying by policy context, population, practice, providers and individual patient needs. Understanding how the design of MDT-care can affect continuity of primary care is critical when making design choices.

MDT also offered some opportunities for *care coordination* through specific coordination roles that may operate within the primary care setting^23^ or help navigate outside of primary care such as other social support services. However, alternative providers coordinating care were not always well received. For example, in MDT-care, patients had concerns about accuracy and confidentiality of information in shared records^35^—this is also identified in the patient safety literature.^60^ Several studies highlighted patients’ preference for relational continuity with and coordination by their own General Practitioner^15^^,^^22^^,^^23^^,^^26^^,^^44^—a preference also supported by General Practitioners.^45^^,^^47^

Implementing MDT-care is not straightforward and requires support at the system, organisational, professional and patient level. One design choice relates to whether MDT-care should be practice-wide or focus on specific patient cohorts—and whether this should be decided at a system level or at an organisational level. For example, the broader literature differentiates between planned or unplanned primary care users, associated with chronic and acute conditions. MDT-care may better support planned users where MDT-care is additional, i.e., those managing chronic conditions.^12^^,^^61^ As reported above, MDT-care may be used to provide quicker access to care for acute conditions when resources are limited and patients can be triaged to the appropriate provider. However, patient needs and provider availability are likely to be context or population specific.

This study showed the impact of MDT-care was limited by factors across systems, organisations and professions; consequently, implementing MDT-care must consider the broader systems that enable MDTs to operate in primary care. MDT-care appeared to be more effective when embedded in professions through training; in teams and organisations through leadership and supported by resources and shared patient records; and in systems through policy frameworks, legislation, training, resourcing, workforce availability, and processes and systems. Consideration must also be given to how teams work collaboratively, including when a team member works at a different location or remotely.^62^ Patients also needed to recognise the value of MDT-care, such as quicker access, greater choice, lower cost, or better outcomes—preferably not compromising one for another.^63^^,^^64^ The review highlighted the difficulty in getting MDT models right in terms of optimal size and composition to reflect the context in which services operated (both patient need and workforce availability) and the time required to implement MDT-care. The design of MDT-care must be flexible to be able to respond to the local context and operating environment recognising this may change over time.

Despite the difficulties, there are clear benefits in using MDT-care in population health and chronic disease management if this is an additional service that is well implemented.^65^ This should be mindful of the purpose of primary care—to provide first contact, comprehensive, coordinated and continuous care over the life course. There are also potential benefits from incorporating other health and social care professionals into general practices. How to resource MDTs and optimise their use is challenging. If done poorly, patients may be required to trade off one component of quality health care (such as access or continuity) for another, and any trade-offs may have a negative impact on patient outcomes and the health system overall.^63^^,^^64^ The implementation of MDTs in primary care must be closely monitored to ensure the desired outcomes are achieved—and monitored across the system, organisational, professional and patient levels.

The study has several limitations. First, the review excluded studies published in non-English languages or practice (grey) literature and did not consider why MDTs were being introduced. Second, this review looked at the relationship between continuity of care and MDT in primary care, given continuity of care is likely to lead to better patient outcomes.^5^^,^^7^, ^8^, ^9^ The review did not purposely identify other benefits of MDT in primary care. That said, included studies also identified other outcomes such as increasing comprehensiveness of care, increasing access to care, and providing coordination. Third, MDT-care differed in each study; this variation requires further analysis. Fourth, the search terms focused on MDT-care and the addition of different professions to primary care. It did not include extended ‘scope of practice’ or ‘models of care’ which is also of interest to policy makers and researchers. Fifth, the review did not investigate payment models—or whether MDT should be designed to fit funding mechanisms or vice versa. Finally, given the research question and the variation in models of MDT care, this research was not able to examine clinical outcomes. Future research should also measure clinical outcomes from different models of MDT-care (compared to non-MDT-care) and their cost-effectiveness, understand the time between implementation and outcomes observed to manage expectations from policy reform, and investigate whether the uptake of MDT varies in regional/rural areas compared to metropolitan areas.

The introduction of MDTs provides an opportunity to strengthen primary care by providing services beyond what a General Practitioner can offer alone—particularly in preventative care and chronic disease management—with General Practitioners remaining an anchor or retaining overall responsibility. The introduction of MDTs may be beneficial where there is a care deficit—such as in preventative care or the comprehensive management of chronic diseases—and where the MDT model addresses that deficit. However, MDTs can undermine continuity of care with a General Practitioner, through task shifting or seeing alternate providers, if not implemented with caution—this is concerning given the relation between continuity of care and quality health care. Continuity of care is most likely to suffer in larger MDTs and potentially exacerbate service fragmentation for patients leading to the need for more care navigators and coordinators. The preconditions required for successful reforms should be considered when introducing MDT-care. Policy change alone is insufficient to realise the benefits of MDT-care; policy needs to be supported by organisations and professions, be made attractive to patients, and requires resourcing across all levels. Any reforms should be closely monitored to understand how MDT-care is used and what this achieves for patients. Research is required to understand the best use of MDT-care in general practice, specifically if and how MDT-care benefits continuity of care, how MDT-care affects clinical outcomes for specific cohorts, whether MDT-care reduces pressure on the health system, and whether this policy is practical and cost-effective long-term.

## Contributors

Conceptualization SB, LA, MW, MK; Data curation SB, JL; Formal analysis SB, JL; Funding acquisition (see acknowledgements); Investigation SB; Methodology SB, JL; Project administration SB; Resources N/A; Software N/A; Supervision LA, MK, MW; Validation SB, LA, JL; Visualization SB, LA; Writing—original draft SB, LA; Writing—review & editing LA, SB, MK, JL, MW.

SB and JL accessed and verified the underlying data. Final approval for the version published has been provided by all authors. All authors agree to be accountable for all aspects of the work.

## Data sharing statement

No new data was generated during this study. This scoping review followed a published protocol on the OSF registry and the Preferred Reporting Items for Systematic Reviews and Meta-Analyses (PRISMA) Extension for Scoping Reviews. The search strategy is included in Appendix pp 1-2. A summary of the data is presented in this paper, along with references to the original source.

## Declaration of interests

MW is the current President of the Royal Australian College of General Practitioners. LA declares an academic contract at the London School of Health and Tropical Medicine paid for by the Peak Vision Foundation, part-time public health and primary care consultancy for the World Bank and WHO, is the Managing Director of Healthier Systems Ltd (global public health consulting company), and declares honorariums from Oxford University Press and CISDI Indonesia (public health conference speaker).
