# Supplementary material for: Can multidisciplinary teams improve the quality of primary care? A scoping review
Source: eClinicalMedicine. 2025 Sep 12;88:103497. doi: 10.1016/j.eclinm.2025.103497 (PMC12572791; doi:10.1016/j.eclinm.2025.103497)
Supplement: Appendix [file mmc1.docx]

Can multidisciplinary teams improve the quality of primary care: A scoping review

Appendix

1. Screening process for studies to be considered eligible for the review

**Types of studies**

1. Research studies (single case)
2. Comparative research studies (two or more cases)
3. Systematic reviews
4. Meta-analysis

**Exclusion criteria**

1. Opinion pieces, conference abstracts, correspondence and editorials

**Types of participants**

1. Patients, and or
2. General practice / primary health care organisations?

**Types of interventions**

1. Introduction of non-General Practitioner primary care providers into a practice – this includes but is not limited to physician assistants, nurse practitioners and practice nurses, paramedics, non-dispensing chemists, psychologists, physiotherapists, dieticians, health educators, and social workers.

**Types of outcome measures**

1. Access to primary care
2. Continuity of care
3. Quality of care
4. Preventative care
5. Health outcomes
6. Patient satisfaction
7. Emergency department presentations and hospitalisations
8. Information continuity
9. Workforce availability
10. Practice composition
11. Workforce satisfaction
12. Cost savings
13. Provider behaviour

2. Search terms for review

{‘primary care’ OR ‘general practice’ OR ‘primary health care’ OR ‘primary healthcare’ OR ‘GP’ OR ‘general practitioner’ OR ‘family physician’ OR ‘general practice nurse’ OR ‘primary care nurse’}

AND

{‘multi-disciplinary’ OR ‘multidisciplinary’ OR ‘inter-disciplinary’ OR ‘interdisciplinary’ OR ‘inter-professional’ OR ‘interprofessional’ OR ‘collaborative practice’ OR ‘team-based care’ OR ‘teambased care’}

AND

{‘continuity of care’ OR ‘care continuity’ OR ‘fragment*’}

IN

{Title Abstract Keyword}.
